# Nitrogen-Doped Titanium Dioxide Nanoparticles Modified by an Electron Beam for Improving Human Breast Cancer Detection by Raman Spectroscopy: A Preliminary Study

**DOI:** 10.3390/diagnostics10100757

**Published:** 2020-09-26

**Authors:** Jakub Surmacki

**Affiliations:** Laboratory of Laser Molecular Spectroscopy, Institute of Applied Radiation Chemistry, Lodz University of Technology, Wroblewskiego 15, 93-590 Lodz, Poland; jakub.surmacki@p.lodz.pl; Tel.: +48-426313188

**Keywords:** human breast cancer, nitrogen-doped titanium dioxide, electron beam irradiation, Raman spectroscopy

## Abstract

Titanium dioxide (TiO_2_) is commonly used as a pigment in paints, paper products, polymer compositions, and cosmetic products, and even as a food additive or drug coating material. In recent times, it has also been used in photovoltaic cells, semiconductors, biomedical devices, and air purification. In this paper, the potential application of nitrogen-doped TiO_2_ nanoparticles modified by an electron beam for improving human breast cancer detection by Raman spectroscopy is presented. Raman spectroscopy (RS) is a promising noninvasive analytical technique in cancer detection that enables us to retrieve a molecular signature of the biochemical composition of cancerous tissue. However, RS still has some challenges in signal detection, mainly related to strong concurrent background fluorescence from the analyzed tissue. The Raman signal scattering is several orders of magnitude smaller than the fluorescence intensity, and strong fluorescence masks a much weaker Raman signal. The Raman results demonstrate that the N-doped TiO_2_ electron beam-irradiated nanoparticles amplify the Raman scattering. The intrinsic properties of the adsorbed molecules from human breast tissue and the surface properties of the N-doped TiO_2_ electron beam-irradiated nanoparticles (the excited electron–hole pair at the surface) have a significant effect on the enhanced Raman signal intensity.

## 1. Introduction

Different spectroscopic techniques have been used to characterize human breast cancer tissue. Techniques such as magnetic resonance imaging (MRI) [1], infrared spectroscopy [2,3,4], and Raman spectroscopy [5,6,7,8,9,10,11,12,13,14,15] have been proven to be powerful methods in characterizing and understanding breast tissue. From a chemical point of view, biological specimens consist of complex mixtures of heterogeneous classes of molecules (e.g., water, lipids, proteins, carbohydrates, and nucleic acids).

Over the last decade, research has intensified in the area of detecting molecular changes from benign to neoplastic tissue. Currently, a low concentration of tumor biomarkers is still undetectable in an early-stage tumor. Here, an optical method of Raman spectroscopy is presented in order to allow the detection of cancerous changes in the human breast using N-doped TiO_2_ nanoparticles modified by electron beam irradiation. The advantages of the Raman technique are its high specificity and its versatility. It is a nondestructive method and requires, in general, only minimal or no sample preparation. Frozen biological specimens with thicknesses of as low as less than 10 µm can be analyzed. However, this optical method has two difficulties: first, it is not easily applicable to materials that exhibit fluorescence, and second, it has inherently poor signal-to-noise ratio. There are a few Raman signal enhancement mechanisms, which have been described in previous publications [16,17,18,19,20,21]. The inelastic light scattering process can be enhanced when the laser is within the molecular absorption bands of the sample. Excitation of this type is in resonance with the electronic transition and yields Raman scattering that is resonance-enhanced [22]. The second mechanism, electromagnetic enhancement, occurs when the incident laser light excites surface plasmons (electrons at the metal surface that collectively oscillate upon excitation), thereby creating an electromagnetic field extending up to 20 nm from the metallic substrate and enhancing Raman signals of exposed molecules [19]. The third mechanism is charge transfer, which transfers electrons between the analyte and metal/metal oxide semiconductor surface when the analyte directly contacts the surface.

In classical surface-enhanced Raman scattering (SERS) experiments, the metallic substrate is made of gold, silver, or copper metal. In this research, semiconductor nanoparticles (N-TiO_2_) modified by electron beam irradiation are used. Pristine titanium dioxide (TiO_2_) has been commonly used as a pigment in paint formulations, paper products, polymer compositions, and semiconductors in photovoltaic cells. Moreover, TiO_2_ has found applications in cosmetic products (e.g., sunscreen, toothpaste), food (e.g., chewing gum, confectionery such as when added to icing sugar), and cancer drugs (as a coating material in tablets with tamoxifen).

In this paper, a new approach for breast cancer diagnosis using N-doped TiO_2_ nanoparticles modified by an electron beam to enhance the Raman signal intensity is presented.

## 2. Materials and Methods 

### 2.1. Chemical and Sample Preparation

All studies and procedures involving human tissue were carried out according to a protocol approved by the institutional Bioethical Committee at the Medical University of Lodz, Poland (No. RNN/31/11/KE). Tissue samples were collected from freshly excised surgical specimens. We used fresh bulk tissue samples and cryosectioned slices from the tumor mass and the tissue from the safety margin outside of the tumor mass obtained during breast surgery. The histological analysis was performed by professional medical doctors, board certified as pathologists, from the Medical University of Lodz, Department of Pathology, Chair of Oncology according to the standard histology protocols.

The bulk tissues or 6 µm cryosectioned slices of infiltrating ductal carcinoma (G2) without staining were closed in a glass vial or mounted on a BaF_2_ window for Raman spectroscopy investigation, respectively. First, pure samples were analyzed, and then the N-doped TiO_2_ nanoparticles modified by electron beam were added. N-doped TiO_2_ nanoparticles were manufactured by the methods described in our previous work [23]. Briefly, commercially available TiO_2_ P25 Degussa (80%:20% mixture of anatase and rutile; 20 nm) was doped with nitrogen by using the wet impregnation method: 5 g of TiO_2_ powder was sustained in 50 mL of urea ((NH_2_)_2_CO) water solution, stirred, and left under mild conditions for 2 weeks until the water evaporated. The urea concentration in solution was such that the atomic ratio of N:Ti in suspension was 1:1. The dried N-doped powder was irradiated with an electron beam (EB) from the linear accelerator ELU-6 MeV (USSR) at ambient temperature. The electron beam irradiation was performed in a continuous regime under the following conditions: pulse frequency = 20 Hz, pulse period = 4 ls, dose rate = 5.5 kGy/min. The total absorbed dose was 500 kGy.

### 2.2. Raman Measurements

The Raman spectra were recorded with a Ramanor U1000 (Horiba, JobinYvon) and a Spectra-Physics 2017-04S argon-ion laser operating at 514 nm with an output power of 52 mW (with a sample of 17 mW), a scanning step of 2 cm^−1^, and an integration time of 0.5 s. The Raman spectrometer Ramanor U1000 was equipped with a macrochamber that allows Raman scattering collection at 90° geometry. The laser spot was d = 500 µm in diameter.

### 2.3. UV-Vis Absorption Measurements

UV-Vis absorption spectra were recorded from powders suspended in OM 100 silicone oil and treated with 35 kHz ultrasound using a Bandelin Sonorex Digitec cleaner for 3 h. After that, treatment samples were centrifuged at around 4000 RPM for 10 minutes. The received supernatants were then easily separated from the samples using simple syringes, placed in 5 mm quartz cuvettes, and analyzed using a Perkin-Elmer 750 spectrometer.

## 3. Results

**Figure 1:**
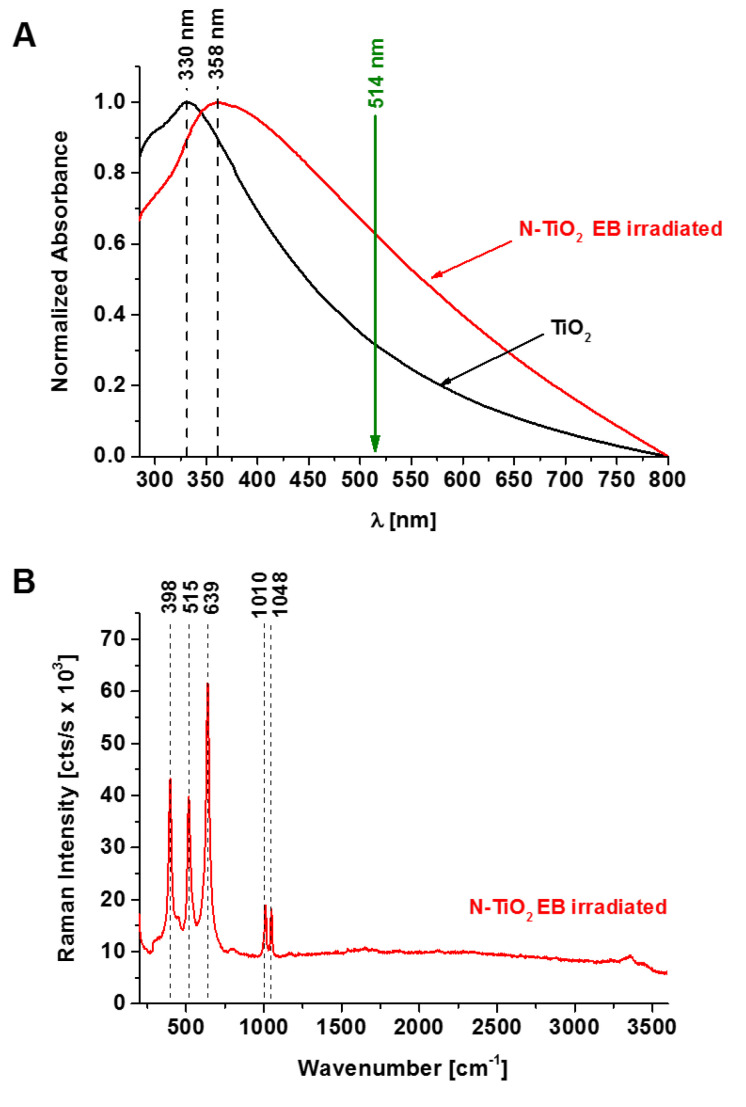
Electronic absorption (**A**) and Raman (**B**) spectra of N-doped TiO_2_ (N:Ti =1:1) nanoparticles electron beam-irradiated with a dose of 500 kGy.

Before we present the results of the Raman spectroscopy for infiltrating ductal carcinoma (tissue sample from patient no. P89 from a database of over 250 patients) with and without the presence of nanoparticles, we first focus on the analysis of N-doped TiO_2_ electron beam (EB)-irradiated nanoparticles (NPs) by UV-Vis and Raman spectroscopy. Figure 1 shows the UV-Vis absorption and Raman spectra of N-doped TiO_2_ (N:Ti = 1:1) NPs EB-irradiated with a dose of 500 kGy. One can see in Figure 1A that an absorption band of TiO_2_ is shifted to the visible region upon N-doping, where efficient resonance conditions of Raman scattering for the excitation with 514 nm are present. A detailed description of the manufacturing and analysis of N-doped TiO_2_ NPs can be found in our previous work [23]. The Raman spectrum of N-doped TiO_2_ EB-irradiated NPs presented in Figure 1B shows characteristic bands at 398, 515, 639, 1010, and 1048 cm^−1^.

**Figure 2:**
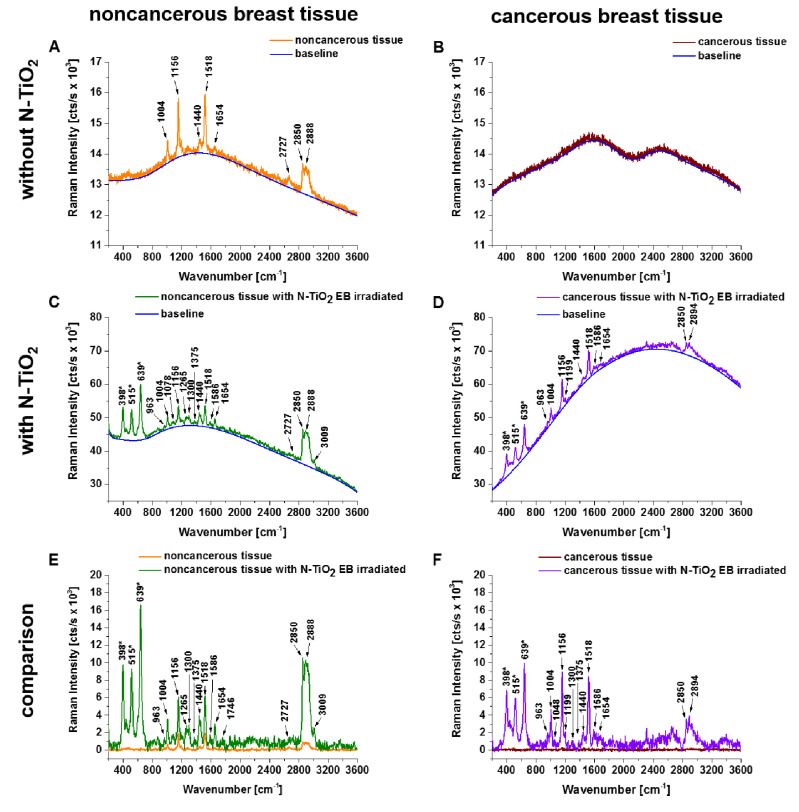
Raman spectra of infiltrating ductal carcinoma (bulk tissue) with and without the addition of N-doped TiO_2_ (N:Ti = 1:1) nanoparticles electron beam-irradiated with a dose of 500 kGy. Subsets (**E**) and (**F**) show a comparison of Raman spectra after background subtraction marked in blue lines on raw Raman spectra (**A**–**D**). Raman bands attributed to N-doped TiO_2_ electron beam (EB)-irradiated nanoparticles (NPs) are marked with an asterisk (*).

**Figure 3:**
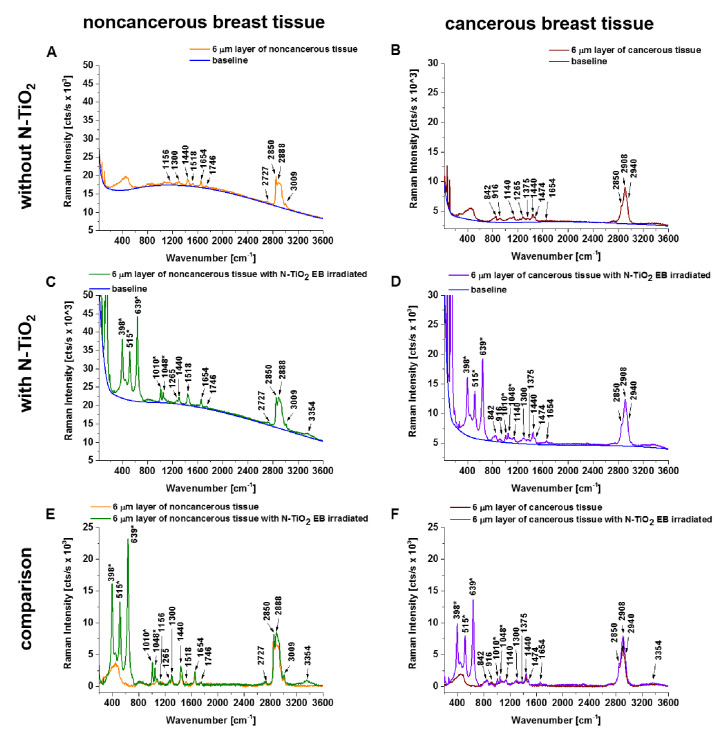
Raman spectra of infiltrating ductal carcinoma (6 µm slice) with and without the addition of N-doped TiO_2_ (N:Ti = 1:1) nanoparticles electron beam-irradiated with a dose of 500 kGy. Subsets (**E**) and (**F**) show a comparison of Raman spectra after background subtraction marked in blue lines on raw Raman spectra (**A**–**D**). Raman bands attributed to N-doped TiO_2_ EB-irradiated NPs are marked with an asterisk (*).

In a second step, we recorded and compared spectra from bulk (Figure 2) and 6 µm slices (Figure 3) of human breast noncancerous and cancerous tissue with and without the addition of N-doped TiO_2_ EB-irradiated NPs. Raman spectra with background fluorescence subtracted allow us to better appraise the effect of enhancement produced by N-doped TiO_2_ EB-irradiated NPs.

Figure 2 shows the Raman spectra of infiltrating ductal carcinoma (bulk tissue) with and without the addition of N-doped TiO_2_ EB-irradiated NPs. The Raman spectra of noncancerous bulk tissue without and with addition of N-doped TiO_2_ EB-irradiated NPs are dominated by spectral bands at 1004, 1156, 1265, 1440, 1518, 1654, 1746, 2727, 2850, 2888, and 3009 cm^−1^ and 398, 515, 639, 842, 963, 1004, 1010, 1048, 1078, 1156, 1199, 1265, 1300, 1375, 1440, 1474, 1518, 1586, 1654, 1746, 2727, 2850, 2888, and 3009 cm^−1^, respectively. Meanwhile, the spectrum of cancerous bulk tissue with the addition of N-doped TiO_2_ EB-irradiated NPs is dominated by bands at 398, 515, 639, 842, 916, 963, 1004, 1010, 1048, 1156, 1199, 1265, 1300, 1375, 1440, 1474, 1518, 1586, 1654, 2850, and 2894 cm^−1^. The Raman spectrum of cancerous bulk tissue reveals an absence of any Raman bands, and only strong fluorescence was observed.

The Raman spectra of 6 µm slices of noncancerous tissue without and with addition of N-doped TiO_2_ EB NPs are dominated by spectral bands at 842, 916, 1004, 1078, 1156, 1265, 1300, 1440, 1518, 1654, 1746, 2727, 2850, 2888, and 3009 cm^−1^ and 398, 515, 639, 842, 916, 963, 1004, 1010, 1048, 1078, 1265, 1300, 1440, 1474, 1654, 1746, 2727, 2850, 2888, 3009, and 3354 cm^−1^, respectively. Meanwhile, the 6 µm slices of cancerous tissue without and with addition of N-doped TiO_2_ EB NPs are dominated by bands at 842, 916, 963, 1004, 1140, 1199, 1265, 1300, 1440, 1474, 1586, 1654, 2850, 2908, and 2940 cm^−1^ and 398, 515, 639, 842, 916, 963, 1004, 1010, 1048, 1140, 1199, 1300, 1375, 1440, 1474, 1586, 1654, 2850, 2908, 2940, and 3354 cm^−1^, respectively.

Detailed peak allocations for carotenoids, lipids, carbohydrates, DNA, and proteins together with an indication of the observed changes with the presence or absence of N-doped TiO_2_ EB-irradiated NPs are shown in Table 1.

**Figure 4:**
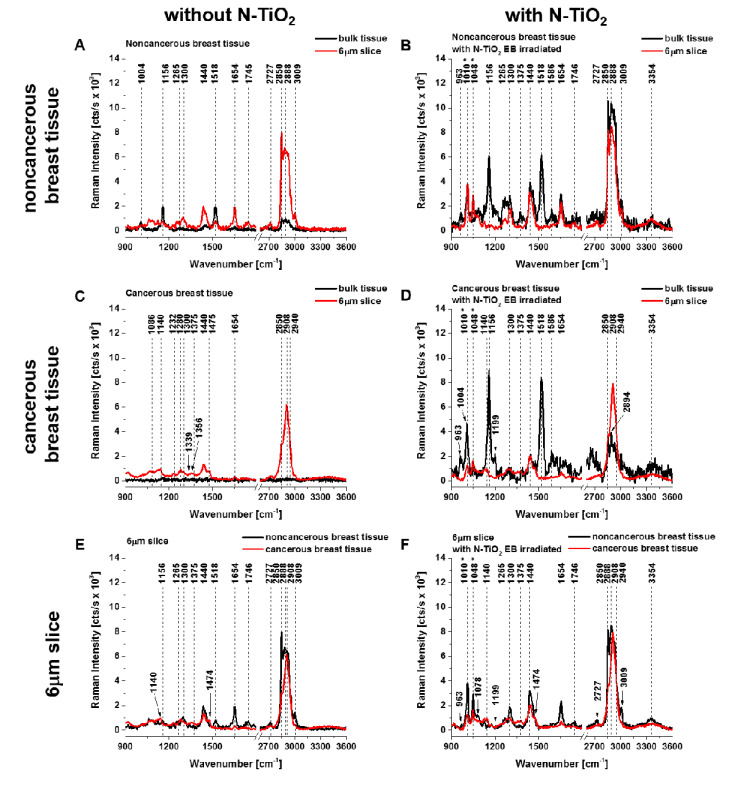
Comparison of Raman spectra for bulk tissues (**A**–**D**) and 6 µm slices (**E**,**F**) of infiltrating ductal carcinoma with (**B**,**D**,**F**) and without (**A**,**C**,**E**) addition of N-doped TiO_2_ (N:Ti = 1:1) nanoparticles electron beam-irradiated with a dose of 500 kGy. Raman spectra are presented after background subtraction. Raman bands attributed to N-doped TiO_2_ EB-irradiated NPs are marked with an asterisk (*).

Figure 4 illustrates a comparison of background-subtracted Raman spectra for bulk tissues and 6 µm slices of noncancerous and infiltrating ductal carcinoma with and without the addition of N-doped TiO_2_ EB-irradiated NPs. The Raman spectra of 6 µm tissue slices reveal much better spectral quality compared to the Raman spectra of bulk tissues. It should be noted that by reducing the sample size to 6 µm, we reduced the number of molecules that emit fluorescence.

## 4. Discussion

The main aim of this preliminary work was to apply Raman spectroscopy as a noninvasive and nondestructive tool for probing human breast tissue in the presence and absence of N-doped TiO_2_ EB-irradiated nanoparticles.

The dominant modes in the Raman spectrum of N-doped TiO_2_ EB NPs presented in Figure 1B can be assigned to the Raman active modes of the anatase crystal: 398 (B1g(1)), 515 (A1g, B1g(2)), and 638 cm^−1^ (Eg(3)). The Raman peaks at 1010 and 1048 cm^−1^ originate from the bending mode (N–H) and new species generated by EB irradiation due to interactions with the polymorphic crystalline structure of TiO_2_ mixed with N-dopants [23], respectively.

The presented preliminary work shows that important differences between the noncancerous and cancerous human breast tissue were found in regions characteristic of vibrations of carotenoids (Raman bands at 1156 and 1518 cm^−1^), lipids (1078, 1300, 1440, 1654, 1746, 2850, 2888, and 3009 cm^−1^), and proteins (842, 1004, 1265, 1586, 1654, 2908, and 2940 cm^−1^) (Figure 4). Moreover, for noncancerous and cancerous tissue the effects of enhancement produced by N-doped TiO_2_ EB NPs, in bulk tissue and 6 µm slices of tissue, in comparison to results without the addition of these NPs, are 8.4 and 4.9 times higher and 15.4 and 1.4 times higher, respectively (calculated as the total integrated intensity of whole background-subtracted Raman spectra presented in Figure 2E,F and Figure 3E,F; noncancerous tissue: bulk (w/o NT) = 440,958 cts/s, bulk (with NT) = 3,708,312 cts/s, 6 µm slice (w/o NT) = 1,644,266 cts/s, 6 µm slice (with NT) = 2,167,831 cts/s; cancerous tissue: bulk (w/o NT) = 197,437 cts/s, bulk (with NT) = 3,033,796 cts/s, 6 µm slice (w/o NT) = 1,188,652 cts/s, 6 µm slice (with NT) = 1,658,370 cts/s). We believe that the explanation for the observed Raman signal enhancement might lie in the N-doped TiO_2_ forms; thus, we used electron–hole pair theory for the explanation [23,27]. The TiO_2_ forms belonged to the n-type semiconductor, which contained many electron–hole (e–h) pairs. The energy gap restricted the transfer of electron–hole pairs when it was excited by the photon. The excitation of electron–hole pairs at the surface of TiO_2_ was possibly transferred via σ–π coordinate bonds and electrostatic force fields between N and Ti to excitations of the adsorbed molecules from breast tissue samples, especially from carotenoids and lipids. The excited adsorbed molecules created molecular fluctuation in the interim, thus leading to the enhancement of Raman scattering. On the other hand, electrons can be transferred from excited states of carotenoids to the conduction band of semiconductor particles and cause enhanced photodegradation and photoisomerization of carotenoids, depending on the properties of both the carotenoids and the semiconductor [28]. It is interesting to note that the photocatalytic reduction of carotenoids was observed only for 6 µm human breast tissue slices (Figure 4) in comparison to bulk tissue samples.

Polyakov et al. showed that the observed enhancement of the photocatalytic efficiency for carotenoid complexes with TiO_2_, as measured by the quantum yield of the desired spin adducts, arises specifically from a decrease in the rate constant for the back electron transfer to the carotenoid radical cation. Polyakov’s results are important for a variety of TiO_2_ applications, namely, photodynamic therapy and the design of artificial light-harvesting, photo-redox, and catalytic devices [29].

Aswini et al. evaluated the cytotoxicity of TiO_2_ nanoparticles against human lung carcinoma cells (A549 cell line). They observed anti-lung cancer activity of TiO_2_ nanoparticles at an IC50 value of 53.65 µg/mL. They proposed a possible mechanism involved in the cancer cell death: ROS (reactive oxygen species) play the main important role in eukaryotic cell death by TiO_2_ nanoparticles. ROS originate from environmental irritants, cellular sources mainly in mitochondria, and NADPH oxidase [30]. This anti-cancer activity might be associated with direct effects of NPs on cells, stimulating the production of free radicals and other oxidants. However, Fu et al. showed that nano-TiO_2_ increased ROS production in UV-exposed cells [31]. Furthermore, NP-derived oxidative stress in vivo may involve mitochondria or NAD(P)H oxidase. Highly reactive hydroxyl acts as a powerful oxidant, resulting in oxidative DNA damage to both single- and double-stranded DNA [30]. Fujiwara et al. found that the oxidative stress produced in response to administration of 6 nm anatase titanium dioxide nanoparticles (on mouse lung and colon cancer cells) was enhanced by high glucose concentrations, acidic pH, hypoxia, high temperature, and the presence of advanced glycation end products [32]. 

Further studies should be performed to determine the anti-tumor properties of N-doped TiO_2_ EB-irradiated nanoparticles and their application in photodynamic therapy.

## 5. Conclusions

In conclusion, our previous study showed that Raman spectroscopy might be a rapid and highly sensitive detection tool for breast cancer diagnosis [11,12,13]. The presented findings are consistent with those of our previous work and show that the application of N-doped TiO_2_ EB-irradiated NPs on breast cancer tissue improves the detection of Raman signals (enhancing it by up to 15.4 times), thus increasing the applicability of this technique in the analysis of intraoperative specimens. Further in vitro studies should be carried out to determine the anticancer properties of N-doped TiO_2_ EB-irradiated nanoparticles on cellular activities (e.g., life-cycle processes) and their photosensitizing properties in photodynamic therapy application.

## Figures and Tables

**Table 1:** Peak allocations for carotenoids, lipids, carbohydrates, DNA, and proteins [12,24,25,26] together with an indication of the observed changes with and without the addition of N-doped TiO_2_ electron beam-irradiated nanoparticles (NPs).

Frequency (cm^−1^)	Assignment	Noncancerous Tissue				Cancerous Tissue			
		Bulk Tissue		6 µm Slice		Bulk Tissue		6 µm Slice	
		w/o	with NPs	w/o	with NPs	w/o	with NPs	w/o	with NPs
398	N-TiO_2_ EB	-	+	-	+	-	+	-	+
515	N-TiO_2_ EB	-	+	-	+	-	+	-	+
639	N-TiO2 EB	-	+	-	+	-	+	-	+
842	Tryptophan, proteins	-	+	+	+	-	+	+	+
916	Proline, hydroxyproline, glycogen, lactic acid	-	-	+	+	-	+	+	+
963	C-O deoxyribose, C-C DNA	-	+	-	+	-	+	+	+
1004	Phenylalanine, proteins, and carotenoids, CH_3_ rocking coupled with C-C stretching	+	+	+	+	-	+	+	+
1010	N-TiO_2_ EB	-	+	-	+	-	+	-	+
1048	N-TiO_2_ EB	-	+	-	+	-	+	-	+
1078	Phospholipids, stretching (C-C) or stretching (C-O)	-	+	+	+	-	-	-	-
1140	Adenine (ring breathing modes of the DNA/RNA bases)	-	-	-	-	-	-	+	+
1156	Carotenoids, C-C stretching	+	+	+	-	-	+	-	-
1199	Nucleic acids and phosphates (P=O)	-	+	-	-	-	+	+	+
1265	Phospholipids, PO_2_^−^ antisymmetric stretching, =C-H in plane deformation, Amide III (of proteins in -helix conformation)	+	+	+	+	-	+	-	-
1300	Lipids, phospholipids, H-C= deformation	-	+	+	+	-	+	+	+
1375	Proteins, C C-H bending mixed with C-N stretching and N-H in plane bending, adenine (ring breathing modes of the DNA/RNA bases)	-	+	-	-	-	+	-	+
1440	Lipids, CH_2_ and CH_3_ deformation vibrations	+	+	+	+	-	+	+	+
1474	DNA, ring breathing mode	-	+	-	+	-	+	+	+
1518	Carotenoids, C=C stretching	+	+	+	-	-	+	-	-
1586	Tryptophan, NADH, cytochrome C, C-C stretching, C-H bending	-	+	-	-	-	+	+	+
1654	Lipids and proteins (amide I), C=C stretching	+	+	+	+	-	+	+	+
1746	Lipids, C=O stretching	+	+	+	+	-	-	-	-
2727	Carotenoids, overtone	+	+	+	+	-	-	-	-
2850	Lipids, fatty acids, saturated bonds of lipids, symmetric stretching of CH_2_	+	+	+	+	-	+	+	+
2888	Lipids, fatty acids, (CH_2_)C-H antisymmetric stretching	+	+	+	+	-	-	-	-
2894, 2908	Symmetric stretching of CH_3_	-	-	-	-	-	+	+	+
2940	Proteins, asymmetric stretching of CH_3_, C-H stretching	-	-	-	-	-	-	+	+
3009	Fatty acids, unsaturated bonds of lipids, H -C=C stretching	+	+	+	+	-	-	-	-
3354	Water, DNA, proteins, O-N, O-H stretching	-	-	-	+	-	-	-	+
